# A Systematized Review of UTI Management Protocol for Long-Term Care Residents

**DOI:** 10.1093/geroni/igaa057.286

**Published:** 2020-12-16

**Authors:** Akanksha Arora, N Ruth Gaskins Little

**Affiliations:** East Carolina University, Greenville, North Carolina, United States

## Abstract

Clinical acuity for the elderly population has significantly intensified. This population is at heightened risk of infection, especially urinary tract infections (UTIs). Urinary tract infections exist as a clinical leviathan in the long-term care (LTC) industry and, despite its high prevalence rates, there exists a dearth of research on management protocol. The following review aims to summarize the current literature, identify, and comparatively analyze the current UTI management guidelines among LTC residents to guide provider leadership development of standards to prevent UTIs. The Joanna Briggs Institute’s approach to systematic reviews was implemented to search the following databases: ProQuest, PubMed, CINAHL, and MEDLINE. 538 citations were assessed, with 32 articles included in the review. Inclusion criteria comprised of clinical trial studies, a time frame of 2005 to current, and no restriction on the study country/region. Key results were collected and analyzed using a data extraction tool. Study findings show that consistent protocols are not followed by licensed staff to prevent, diagnose, and treat UTI’s among the elderly residing in long term care facilities. Inappropriate use of antibiotics is problematic due to the lack of specific practice guidelines for testing, diagnosis and, treatment. Studies implementing (1) successful clinical management strategies (i.e. antibiotic initiation, urinalysis frequency) and (2) facility administrative strategies (i.e. incontinence nurse specialist, DON/nurse leadership education) are needed to establish gold standard practice guidelines for the LTC industry.

